# mSphere of Influence: The challenge of screening for essential protein kinases in *Trypanosoma cruzi*

**DOI:** 10.1128/msphere.00598-24

**Published:** 2025-01-28

**Authors:** Miguel A. Chiurillo

**Affiliations:** 1 Department of Biological Sciences, University of Cincinnati 2514 , Cincinnati, Ohio, USA; University of Georgia, Athens, Georgia, USA

**Keywords:** *Trypanosoma cruzi*, kinoma, bar-seq

## Abstract

Miguel Chiurillo works in the field of protein kinases, studying their role in cell signaling and cell cycle progression in *Trypanosoma cruzi*. In this mSphere of Influence article, he reflects on how the research articles “Systematic functional analysis of *Leishmania* protein kinases identifies regulators of differentiation or survival” by Baker et al. and “Screening the *Toxoplasma* kinome with high throughput tagging identifies a regulator of invasion and egress” by Smith et al. made an impact on his understanding of the complexity of the *Trypanosoma cruzi* kinome and the challenges to unravel it.

## COMMENTARY

Protein kinases (PK) are key regulators of signal transduction pathways in eukaryotes. These enzymes catalyze the transfer of phosphate groups from high-energy phosphate-donating molecules to specific substrates. Protein phosphorylation has been involved in several pivotal roles in protozoan parasites, such as cellular differentiation, cell cycle control, and adaptation to environmental changes (1). *Trypanosoma cruzi*, the etiological agent of Chagas disease, has a reduced kinome compared to its human equivalent (2, 3). However, in these parasites, three eukaryotic PK (ePK) families (GMGC, STE, and NEK) are significantly expanded with respect to human PKs (2). An increasing body of evidence supports the study of PKs in trypanosomatids. First, there are significant differences in their kinome composition compared to mammalian cells, which include low sequence identity and unique PKs with no orthologs in mammals. Second, PKs play a major role in the life cycle of protozoan parasites. Furthermore, less than 2% of the *T. cruzi* kinome has been functionally analyzed using loss-of-function approaches. Finally, there is cumulative evidence showing that PKs are potential drug targets in trypanosomatids (4–6). Large-scale gene screenings in parasitic protozoans were limited to those having RNAi machinery to perform gene down-regulation, such as *Trypanosoma brucei* (7). However, since CRISPR/Cas9 technology has been implemented in many of these organisms, this kind of strategies is now feasible at several scales, from the analysis of a relatively large group of genes, such as functionally related genes, to a genome-wide screening. Moreover, the inclusion of barcode analyses (bar-seq) to CRISPR-based methods is facilitating the simultaneous characterization of a large cohort of mutants (8). In this context, two recent large-scale loss-of-function studies on the kinome of protozoan parasites have caught my attention regarding the possibility of performing simultaneous analysis of multiple PK genes rather than individually studying each one of them in *T. cruzi*. In the first one, Baker et al. (9) applied a kinome-wide gene deletion strategy in *Leishmania mexicana*. Here, the authors followed a similar approach previously used to study the flagellar proteome of *L. mexicana* (8), which combines a CRISPR/Cas9 gene deletion strategy with a next-generation sequencing (NGS) of a unique barcode included in the donor cassette used to induce homologous directed repair. In this large-scale effort, the authors were able to establish the importance of 204 kinases (193 ePKs and 11 atypical PKs) throughout the life cycle of this parasite. Initially, they found 44 essential PK genes for promastigote replication because null mutants could not be obtained for these genes. Subsequently, to establish the role of PKs in differentiation in the life cycle of *Leishmania*, the authors pooled the PK mutants and assessed their relative fitness by bar-seq analysis to determine the relative abundance of each cell line in cultures of procyclic promastigotes, metacyclic promastigotes, axenic amastigotes, amastigotes in macrophages, and amastigotes in the footpads of mice. Authors reported 29 PKs important for amastigotes across at least two of the three experimental arms in which this stage was assessed and additional 15 PKs that showed loss of fitness in mouse infection only. Baker et al. also identified 15 PKs with loss of fitness in the ability to colonize the sand fly vector. In this approach, I found particularly interesting the use of different sub-pools of parasites based on growth profiles, to reduce the effect of competition between mutants and functional compensation in pooled populations.

In a different study, Smith et al. (10) used a NHEJ-deficient *Toxoplasma gondii* strain to systematically examine the kinome of this parasite in a large-scale CRISPR-based approach. In this study, the authors developed a high-throughput tagging (HiT) strategy to endogenously tag PKs with an auxin-inducible degron (mAID) fused to a fluororescent protein (mNeonGreen). The donor DNA also allowed the integration of a gRNA sequence providing a unique barcode to identify each mutant. The system allowed them to assess the subcellular localization of HiT-tagged PKs, as well as their relevance for parasite survival, since gene downregulation was achieved by the addition of indole-3-acetic acid (IAA) for proteasomal degradation of mAID-tagged proteins. Overall, Smith et al. targeted 147 *T. gondii* PKs, of which 127 (82%) had a single gRNA detected by NGS, that were then examined in arrayed and pooled screenings. By examining more than a thousand arrayed clones, these authors assigned 40 PK localizations and identified 15 PKs with defective phenotypes by microscopy. Smith et al. also measured the fitness of tagged alleles by pooling screening, a strategy that offers scalability and is more sensitive than arrayed screenings to determine which PKs contribute to parasite fitness.

Considering the need of using donor DNAs for double-strand break repair in trypanosomatids, where the NHEJ machinery is absent, the generation of bar-seq KO libraries in *T. cruzi* would involve mutants generated individually and barcoded in the process of gene deletion before pooling them in culture. For this purpose, we recently developed an efficient cloning-free T7RNAP/Cas9 strategy that facilitates gene tagging and deletion in *T. cruzi* and is suitable for the inclusion of DNA barcodes to tag genes individually (11). This system could facilitate functional gene analyses at large scale in this parasite. The few studies that have explored the function of PKs in *T. cruzi* have followed a single-gene ablation strategy (12, 13). Therefore, systematic approaches as those followed by Baker et al. and Smith et al. would be useful to visualize the total contributions of individual PKs to parasite fitness. However, the lack of conditional KO strategies or robust inducible downregulation systems in *T. cruzi*, as well as inherent limitations of the model to scale its genetic manipulation, represents important challenges to conduct multigene loss-of-function studies in this parasite. Despite these challenges, both studies (9, 10) have encouraged me to design a promising multigene analysis strategy to investigate the largely unexplored kinome of this parasite at this stage of my research career, with the help of the countless benefits of CRISPR/Cas9 technology.
